# Research and therapeutic status of NSAID-exacerbated respiratory disease: A bibliometric study from 2000 to 2024

**DOI:** 10.1097/MD.0000000000051236

**Published:** 2026-10-30

**Authors:** Chenxi Li, Dayu Guan, Yuan Chen, Yalan Zhang, Ji Wang, Yue Gu, Bowen Zheng, Yuchuan Liu, Xiaoling Zhang, Tao Lu, Yang Shen, Yucheng Yang

**Affiliations:** a Department of Otolaryngology Head and Neck Surgery, The First Affiliated Hospital of Chongqing Medical University, Chongqing, China; b Department of Otolaryngology Head and Neck Surgery, Wushan County People’s Hospital of Chongqing, Chongqing, China.

**Keywords:** bibliometrics, diagnosis, NSAID-exacerbated respiratory disease, treatment, visualization

## Abstract

**Background:**

Nonsteroidal anti-inflammatory drug-exacerbated respiratory disease (NERD) is a chronic inflammatory condition clinically characterized by the triad of asthma, nasal polyps, and hypersensitivity reactions after aspirin ingestion. It is a distinct syndrome that is closely associated with eosinophilic infiltration of respiratory tissues and excessive production of cysteinyl leukotrienes. Research on NERD is characterized by fragmented knowledge, an incomplete understanding of its underlying mechanisms, and a lack of standardized approaches for its diagnosis and treatment. This study systematically summarized the major research directions and advances in identifying hotspots and emerging trends in NERD.

**Methods:**

To address these challenges, we retrieved NERD-related literature from 2000 to 2024 from the Web of Science Core Collection and performed a bibliometric analysis using CiteSpace, VOSviewer, and Bibliometrix to help researchers quickly understand the field’s evolution and research frontiers.

**Results:**

The analysis showed that, over the past 24 years, publication output in this field has grown steadily. Notably, the United States has made the largest contribution. At the institutional level, Jagiellonian University in Poland had the highest number of publications, and Professor Marek Sanak was identified as the most prolific author. The Journal of Allergy and Clinical Immunology was the most frequently cited journal in this field. Keyword co-occurrence and cluster analysis revealed that the current research themes focused on disease pathogenesis and treatment approaches. The journal dual-map overlay analysis further showed that research trends have gradually shifted from molecular biology and genetics to immunology and clinical research.

**Conclusion:**

This study provides important insights into the current status, advances, and emerging trends of NERD.

## 1. Introduction

Nonsteroidal anti-inflammatory drug (NSAID)-exacerbated respiratory disease (NERD), also known as Samter triad, was 1st described by Samter and Beers approximately 5 decades ago. It is characterized by chronic rhinosinusitis with nasal polyps (CRSwNP), moderate-to-severe asthma, and hypersensitivity reactions to NSAIDs such as aspirin. The European Academy of Allergy and Clinical Immunology has recently proposed an updated definition of NERD: “a chronic eosinophilic inflammatory disease of the airways occurring in patients with asthma and/or CRSwNP, whose symptoms are exacerbated by the intake of NSAIDs, including aspirin.”^[1]^ Clinically, NERD manifests as persistent type-2 inflammation of both the upper and lower airways, and after exposure to aspirin or other NSAIDs, patients may develop nasal obstruction, worsening asthma, and ocular pruritus. Some patients also experience extra-respiratory symptoms involving the gastrointestinal system (abdominal pain, nausea, and vomiting) and skin (flushing and pruritus). In most patients, reactions occur after NSAID ingestion across a dose range of approximately 10 to 300 mg, with onset and severity generally related to the administered dose.^[2]^ Prevalence estimates vary substantially across countries and studies: approximately 0.3% to 0.9% in the general population and higher among those with asthma and CRSwNP, approximately 7% and 10% to 21%, respectively.^[3]^ Furthermore, NERD is associated with significantly increased risks of acute asthma exacerbation, hospitalization, and emergency visits. Despite a growing body of work in recent years, research in this field remains fragmented, with an incomplete understanding of the pathophysiology and insufficient standardization of diagnosis and management. Although many studies have addressed the molecular mechanisms, clinical features, and therapeutic strategies, the connections among them are often tenuous, and a unified knowledge framework is lacking. This fragmentation has prevented researchers and clinicians from understanding the field as a whole, identifying key scientific questions, and achieving major breakthroughs.

Bibliometrics is a scholarly approach that combines quantitative and qualitative analyses of the scientific literature to elucidate the knowledge structure and developmental trajectory of a field.^[4,5]^ In 1969, bibliometrics pioneer Alan Pritchard introduced the concept and defined bibliometrics as the application of mathematics and statistical methods that shed light on the processes of written communication, which is generally used to analyze research trends in publications. Bibliometric methods can map distributions, relationships, and clusters within a research domain; help forecast future directions; and, compare the contributions of authors, institutions, countries, and journals. Common visualization tools include VOSviewer and CiteSpace, which are widely used, particularly in the medical sciences, to examine citation impacts, research hotspots, and collaboration networks. Consequently, bibliometrics has become a popular technique for rapidly apprehending the evolution and frontiers of a specific research area and evaluating the quality and trajectory of scholarly output.

The Web of Science Core Collection (WoSCC) indexes a large volume of published research. However, these studies are dispersed and have not been systematically integrated. With NERD drawing increasing attention and related publications proliferating in recent years, to the best of our knowledge, no bibliometric study has focused specifically on NERD. Therefore, a comprehensive synthesis is required to clarify the principal contributors and current landscape, delineate priority research themes, and outline future trends and prospects. This study covers NERD-related literature from 2000 to 2024 and employs bibliometric analyses to construct network maps of authors, countries, institutions, keywords, and journals. Building on these analyses, we systematically summarized the major research directions and advances in identifying hotspots and emerging trends in NERD, thereby providing a more integrated and in-depth understanding of the field and offering useful guidance for researchers and policymakers.

## 2. Methods and materials

### 2.1. Search strategy

We queried the Web of Science database, selecting “Search in: Web of Science Core Collection.” The WoSCC is widely regarded as one of the most authoritative global databases and has become the preferred source for bibliometric analyses. In addition, WoSCC provides complete citation information, which robustly supports subsequent knowledge-mapping analyses. Our search strategy was as follows: topic search (TS) = (Aspirin-Induced Asthmas OR Asthma, Aspirin-Induced OR Asthmas, Aspirin-Induced OR Aspirin-Induced Asthma OR Aspirin-Induced Asthma OR Aspirin-Induced Asthmas OR Asthmas, Aspirin-Induced OR Induced Asthma, Aspirin OR Induced Asthmas, Aspirin OR Aspirin-Induced Asthma Syndrome OR Aspirin-Induced Asthma Syndrome OR Aspirin-Induced Asthma Syndromes OR Asthma Syndrome, Aspirin-Induced OR Asthma Syndromes, Aspirin-Induced OR Syndrome, Aspirin-Induced Asthma OR Syndromes, Aspirin-Induced Asthma OR Asthma, NSAID-induced OR Asthma, NSAID-induced OR Asthmas, NSAID-induced OR NSAID-induced Asthma OR NSAID-induced Asthmas OR NSAID-Exacerbated Respiratory Disease OR Disease, NSAID-Exacerbated Respiratory OR Diseases, NSAID-Exacerbated Respiratory OR NSAID-Exacerbated Respiratory Disease OR NSAID-Exacerbated Respiratory Diseases OR Respiratory Disease, NSAID-Exacerbated OR Respiratory Diseases, NSAID-Exacerbated OR NERD NSAID-Exacerbated Respiratory Disease OR NERD NSAID-Exacerbated Respiratory Disease), limited to publications from January 1, 2000 to January 1, 2025. We included only original research articles and review articles written in English, and excluded other document types and non-English publications. To minimize bias due to periodic database updates, we executed the search and downloaded the data on May 1, 2025. To determine eligibility, 2 reviewers independently screened all titles and abstracts identified by the search strategy; disagreements were resolved through discussion or, when necessary, consultation with an additional expert.

### 2.2. Bibliometric visualization tools and methods

We employed 3 commonly used tools for bibliometric visualization: VOSviewer (version 1.6.18; Centre for Science and Technology Studies, Leiden University), CiteSpace (version 6.4.R1; Drexel University), and Bibliometrix (Department of Economics and Statistics University of Naples Federico II). VOSviewer and Bibliometrix were used to generate network and overlay visualizations for countries, institutions, authors, and journals, whereas CiteSpace and Bibliometrix were used to construct timezone maps and to detect burst keywords. In this study, VOSviewer and CiteSpace were applied to perform co-occurrence analysis, clustering analysis, and timezone visualizations for countries, institutions, authors, journals, and keywords. Burst detection was conducted for countries, authors, and keywords, and a journal dual-map overlay was produced to illustrate citation trajectories across disciplines.

## 3. Results

### 3.1. Literature search results

In this study, a total of 1018 articles were retrieved. After excluding 110 records (conference abstracts, editorials, letters, and book chapters) and 71 non-English articles as per the screening process, 837 articles were finally included. The detailed search procedure is shown in Figure 1. The volume of literature output in a given research field reflects its developmental status, accumulated knowledge, and level of maturity. Over the past 24 years, the number of publications related to NERD has steadily increased (Fig. 2), with 2013 seeing the highest number of publications.

**Figure 1.**
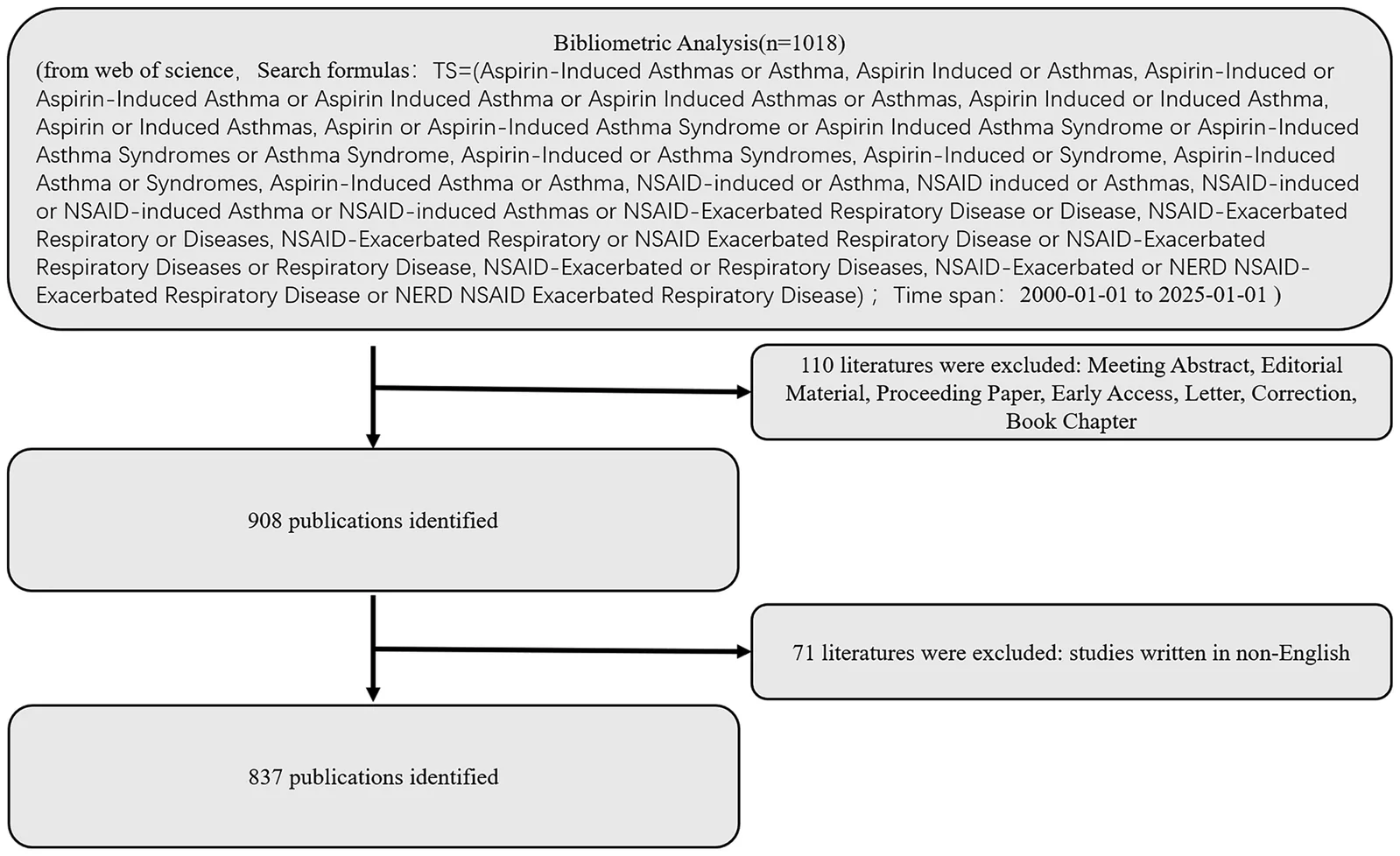
Flowchart of search strategy and data collection. NSAID = nonsteroidal anti-inflammatory drug, TS = topic search.

**Figure 2.**
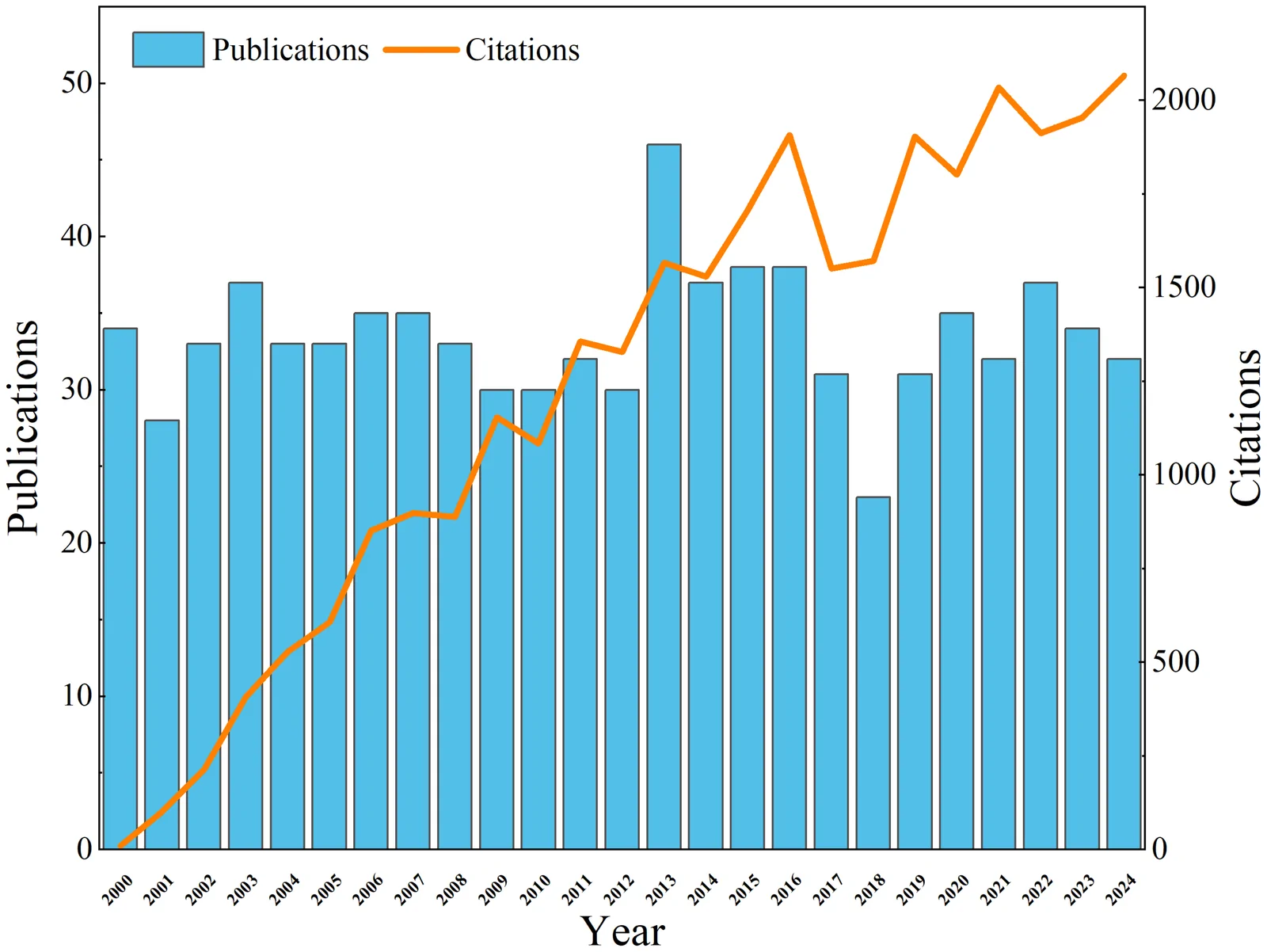
Statistics on the publication and citation trends of NERD-related literature. NERD = nonsteroidal anti-inflammatory drug-exacerbated respiratory disease.

### 3.2. National collaboration analysis

Using VOSviewer (with default thresholds) to analyze the downloaded data, we mapped the publications from 22 different countries and their collaboration relationships (Fig. S1A, Supplemental Digital Content 1), as well as a time-overlay visualization by publication year (Fig. S1B, Supplemental Digital Content 2). The top 10 countries by publication volume are the United States (242 articles), Poland (102), Spain (99), South Korea (83), Japan (67), the United Kingdom (54), Germany (46), Italy (44), Turkey (43), and France (31) (Table 1). Poland and Italy began NERD research relatively early, with their publication surges (citation bursts) occurring around 2000, and they have remained among the world leaders in academic influence. The United States, Spain, and Germany have the highest total number of citations (Table 1). Over the past 24 years, the United States has consistently been the most active country in NERD research and publications (Fig. S1A and C, Supplemental Digital Content 3). This reflects the United States’ global leadership in biomedical research, supported by ample R&D funding and the largest number of publications in top journals.

**Table 1:** Top 10 countries in the field of NERD.

Country	Documents	Citations	Total link strength
The United States of America	242	12,356	123
Poland	102	6620	77
Spain	99	5016	104
South Korea	83	1877	15
Japan	67	2860	25
United Kingdom	54	3631	86
Germany	46	2352	89
Italy	44	2346	68
Turkey	43	1718	26
France	31	1550	81

NERD = nonsteroidal anti-inflammatory drug (NSAID)-exacerbated respiratory disease.

### 3.3. Institution collaboration analysis

The number of publications is an important metric of research strength and a key indicator of an institution’s academic influence. Through the institution collaboration analysis, we identified the most authoritative organizations conducting research on this topic. In total, 24 institutions were mapped, resulting in a collaboration network and a time-overlay visualization (Fig. S2A–C, Supplemental Digital Content 4). The top 10 institutions by publication count are Jagiellonian University (59 articles), Scripps Clinic (51), Brigham & Women’s Hospital (39), Ajou University (38), University of Barcelona (29), Soonchunhyang University (29), Medical University of Lodz (27), Harvard Medical School (25), Harvard University (24), and Sogang University (20) (Table 2). These institutions not only rank highly in publication output but are also at the forefront of NERD research. These institutions form distinct collaboration clusters. Cluster analysis identified 5 major clusters, each marked by a different color corresponding to a geographic region. The red cluster consists of 7 institutions from the USA and Spain (Brigham & Women’s Hospital, Harvard Medical School, Harvard University, University of Barcelona, Hospital Clínic de Barcelona, Northwestern University, and Vanderbilt University); the green cluster comprises 6 institutions in South Korea (Ajou University, Soonchunhyang University, Sogang University, SNP Genetics Inc., Chungbuk National University, and Chonnam National University); and the blue cluster comprises 5 institutions in Poland, Sweden, and the UK (Jagiellonian University, Medical University of Lodz, Karolinska Institute, University of Southampton, and AGH University of Science & Technology). The yellow cluster includes 4 institutions from the USA and Turkey (Scripps Clinic, Scripps Research Institute, Hacettepe University, and Ankara University), and the purple cluster includes 2 institutions in Spain (Infanta Leonor Hospital and University of Extremadura). The wide geographic distribution of these research institutions indicates that NERD has attracted global interest.

**Table 2:** Top 10 institutions in the field of NERD.

Institution	Documents	Citations	Total link strength
Jagiellonian University	59	4857	33
Scripps Research	51	3863	31
Brigham and Women’s Hospital	39	2550	46
Ajou University	38	1178	18
University of Barcelona	29	1802	23
Soonchunhyang University	29	482	60
Medical University of Lodz	27	1583	12
Harvard Medical School	25	1258	32
Harvard University	24	1919	21
Sogang University	20	289	63

NERD = nonsteroidal anti-inflammatory drug (NSAID)-exacerbated respiratory disease.

### 3.4. Author collaboration analysis

Using VOSviewer, we constructed an author collaboration network consisting of 28 authors along with a time-overlay visualization colored by publication year (Fig. S3A, S3B, S3C, Supplemental Digital Content 7). The top 10 most prolific authors in NERD research are Marek Sanak (35 publications), Donald D. Stevenson (32), Choon-Sik Park (27), Tanya M. Laidlaw (26), Miguel Blanca (25), Hae-Sim Park (25), A. Szczeklik (25), Ronald A. Simon (23), Natalia Blanca-López (22), and Andrew A. White (21) (Table 3). In the author collaboration network, Marek Sanak, Donald D. Stevenson, Choon-Sik Park, Tanya M. Laidlaw, and Miguel Blanca each occupy central positions in different clusters, indicating that these authors have been instrumental in advancing NERD research. Figure 3 illustrates the collaboration links between authors, institutions, and countries. Notably, Professor Tanya M. Laidlaw from Brigham and Women’s Hospital (a Harvard Medical School–affiliated hospital in the USA) collaborates with numerous institutions across multiple countries.

**Table 3:** Top 10 authors in the field of NERD.

Author	Documents	Citations	Total link strength
Marek Sanak	35	1690	33
Donald D Stevenson	32	3098	23
Choon-Sik Park	27	258	84
Tanya M. Laidlaw	26	1762	33
Miguel Blanca	25	1773	54
Hae-Sim Park	25	786	25
Andrzej Szczeklik	25	2739	14
Ronald A Simon	23	1438	23
Natalia Blanca-Lopez	22	1332	48
Andrew A White	21	1392	21

NERD = nonsteroidal anti-inflammatory drug (NSAID)-exacerbated respiratory disease.

**Figure 3.**
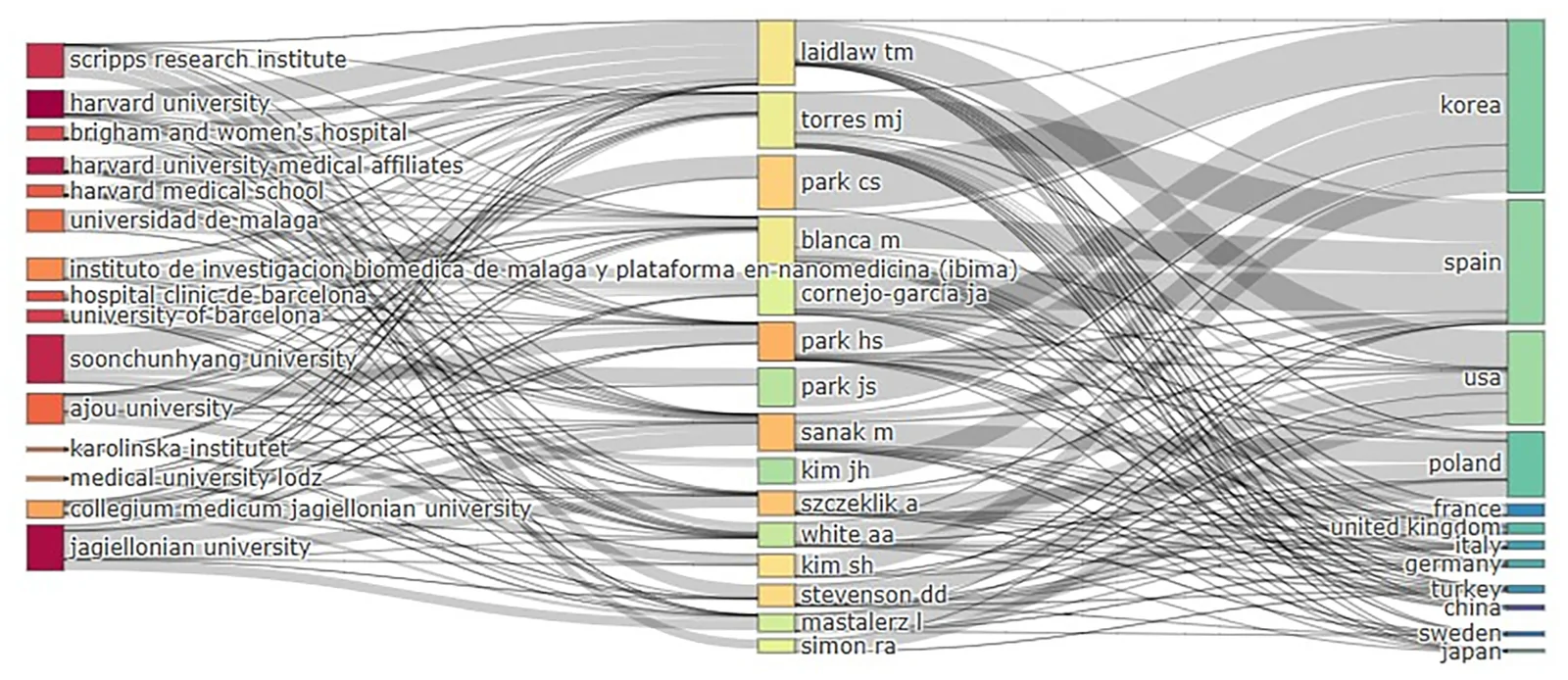
Three-field plot of the NERD domain. Left: institutions; middle: authors; and right: countries. The gray curves denote connection relationships, with curve width indicating the strength of collaboration. NERD = nonsteroidal anti-inflammatory drug (NSAID)-exacerbated respiratory disease.

### 3.5. Journal analysis

Journals are the primary vehicles through which research is disseminated to the scientific community. In terms of journal output, Table 4 summarizes the characteristics of the 10 most prolific journals publishing on NERD; the vast majority are within the fields of allergy and immunology. The journals with the highest numbers of NERD-related publications were Journal of Allergy and Clinical Immunology (65 articles), Allergy (44), Clinical & Experimental Allergy (40), Annals of Allergy, Asthma & Immunology (37), Journal of Allergy and Clinical Immunology: In Practice (34), Journal of Investigational Allergology and Clinical Immunology (23), International Archives of Allergy and Immunology (20), Allergy and Asthma Proceedings (19), Current Allergy and Asthma Reports (19), and Immunology and Allergy Clinics of North America (19). Collectively, these outlets represent, to a considerable extent, the authoritative venues for research in the NERD field. Co-cited references are those cited simultaneously by 2 or more publications. The 10 most frequently co-cited references are listed in Table 5,^[6–15]^ among which clinical studies are the most common type. The single most highly cited reference is a multicenter retrospective clinical study published in 2000 in the European Respiratory Journal.

**Table 4:** Top 10 journals in the field of NERD.

Journal	Documents	Citations	Impact factor (2024)
Journal of Allergy and Clinical Immunology	65	4721	11.2
Allergy	44	2905	12.0
Clinical & Experimental Allergy	40	1815	5.2
Annals of Allergy, Asthma & Immunology	37	1415	4.7
The Journal of Allergy and Clinical Immunology: In Practice	34	1141	6.6
Journal of Investigational Allergology and Clinical Immunology	23	488	4.8
International Archives of Allergy and Immunology	20	469	1.8
Allergy & Asthma Proceedings	19	332	2.2
Current Allergy and Asthma Reports	19	490	4.6
Immunology and Allergy Clinics of North America	19	532	2.2

NERD = nonsteroidal anti-inflammatory drug (NSAID)-exacerbated respiratory disease.

**Table 5:** Top 10 co-cited references in NERD.

Co-cited reference	Citations	Journal	Types
Szczeklik A. DOI: 10.1034/j.1399-3003.2000.016003432.x. 2000.^[6]^	167	Eur Respir J.	Article
Cowburn AS. DOI: 10.1172/JCI620. 1998.^[7]^	151	J Clin Invest.	Article
Szczeklik A. DOI: 10.1067/mai.2003.1487. 2003^[8]^	141	J Allergy Clin Immunol.	Review
Samter M. DOI: 10.7326/0003-4819-68-5-975. 1968.^[9]^	119	Ann Intern Med.	Clinical trial
Christie PE. DOI: 10.1164/ajrccm/143.5_Pt_1.1025. 1991.^[10]^	107	Am Rev Respir Dis.	Clinical trial
Berges-Gimeno MP. DOI: 10.1016/S1081-1206(10)62084-4. 2002.^[11]^	103	Ann Allergy Asthma Immunol.	Article
Nizankowska-Mogilnicka E. DOI: 10.1111/j.1398-9995.2007.01409.x. 2007.^[12]^	103	Allergy.	Review
Szczeklik A. DOI: 10.1016/s0091-6749(99)70106-5. 1999.^[13]^	100	J Allergy Clin Immunol.	Review
Jenkins C. DOI: 10.1136/bmj.328.7437.434. 2004.^[14]^	99	BMJ.	Review
Sousa AR. DOI: 10.1056/NEJMoa013508. 2002.^[15]^	93	N Engl J Med.	Clinical trial

NERD = nonsteroidal anti-inflammatory drug (NSAID)-exacerbated respiratory disease.

### 3.6. Keyword analysis

Keywords concisely capture a paper’s core content and can be used to trace the evolution of research hotspots and trends. We imported the downloaded data into CiteSpace and set the following parameters: time slicing from 2000 to 2024 with a 1-year interval; the selection threshold was Top 2%; and keyword clustering with visualization. The results are shown in Figure S4A, Supplemental Digital Content 10. The 10 most frequent keywords were “aspirin-induced asthma,” “intolerant asthma,” “nasal polyps,” “diagnosis,” “chronic rhinosinusitis,” “asthma,” “nonsteroidal antiinflammatory drugs,” “aspirin-exacerbated respiratory disease,” “intolerance,” and “expression,” indicating the principal foci of research in this domain. The betweenness centrality values of “aspirin-induced asthma,” “intolerant asthma,” “diagnosis,” “nonsteroidal antiinflammatory drugs,” and “expression” all exceeded 0.1, suggesting a bridging, hub-like role that helps connect hotspots and critical turning points (Table 6). Using the log-likelihood ratio algorithm in CiteSpace for keyword clustering, we generated the cluster map shown in Figure S4B, Supplemental Digital Content 11. Four clusters were identified: #0 “aspirin-exacerbated respiratory disease,” #1 “nonsteroidal antiinflammatory drugs,” #2 “diagnosis,” and #3 “leukotriene receptor antagonist.” Figure S4C, Supplemental Digital Content 12 presents the top 20 burst keywords from 2000 to 2024; “chronic rhinosinusitis” exhibited the strongest burst (29.57), followed by “intolerant asthma” (20.22) and “nasal polyps” (17.27). “Provocation tests” is the most recent burst term and also shows a relatively strong burst (6.41). By examining the temporal distribution of clusters in the visualizations in Figure 4A–C, we further delineated the shifting hotspots in NERD research: the focus progressed from inflammatory mechanisms to specific clinical syndromes; broad asthma research branched into distinct phenotypes, with growing attention to comorbidities such as nasal polyps. Figure 4D displays the keyword thematic map produced with bibliometrix: Quadrant I (high centrality, high density) indicates core themes of the field – current priorities and hotspots. Quadrant II (low centrality and high density) encompasses well-developed yet relatively isolated topics that are specialized and narrowly focused. Quadrant III (low centrality and low density) points to emerging or declining themes, for which temporal context is needed to judge trajectory. Quadrant IV (high centrality and low density) reflects foundational themes that are broadly connected to many others but whose internal development remains immature or incomplete.

**Table 6:** Top 20 keywords by frequency in the field of NERD.

Keyword	Count	Centrality	Year
aspirin-induced asthma	161	0.75	2000
intolerant asthma	146	0.13	2004
nasal polyps	83	0.03	2009
diagnosis	79	0.12	2004
chronic rhinosinusitis	77	0.00	2013
asthma	63	0.02	2005
nonsteroidal anti-inflammatory drugs	52	0.19	2003
aspirin-exacerbated respiratory disease	43	0.00	2013
intolerance	42	0.06	2002
expression	38	0.17	2002
aspirin	33	0.09	2002
exacerbated respiratory-disease	28	0.04	2003
pathogenesis	27	0.01	2003
hypersensitivity	23	0.02	2003
prevalence	16	0.00	2013
natural history	15	0.00	2010
challenge	14	0.06	2000
cysteinyl leukotrienes	13	0.01	2008
leukotriene receptor antagonist	12	0.00	2001
provocation tests	10	0.00	2017

NERD = nonsteroidal anti-inflammatory drug (NSAID)-exacerbated respiratory disease.

**Figure 4.**
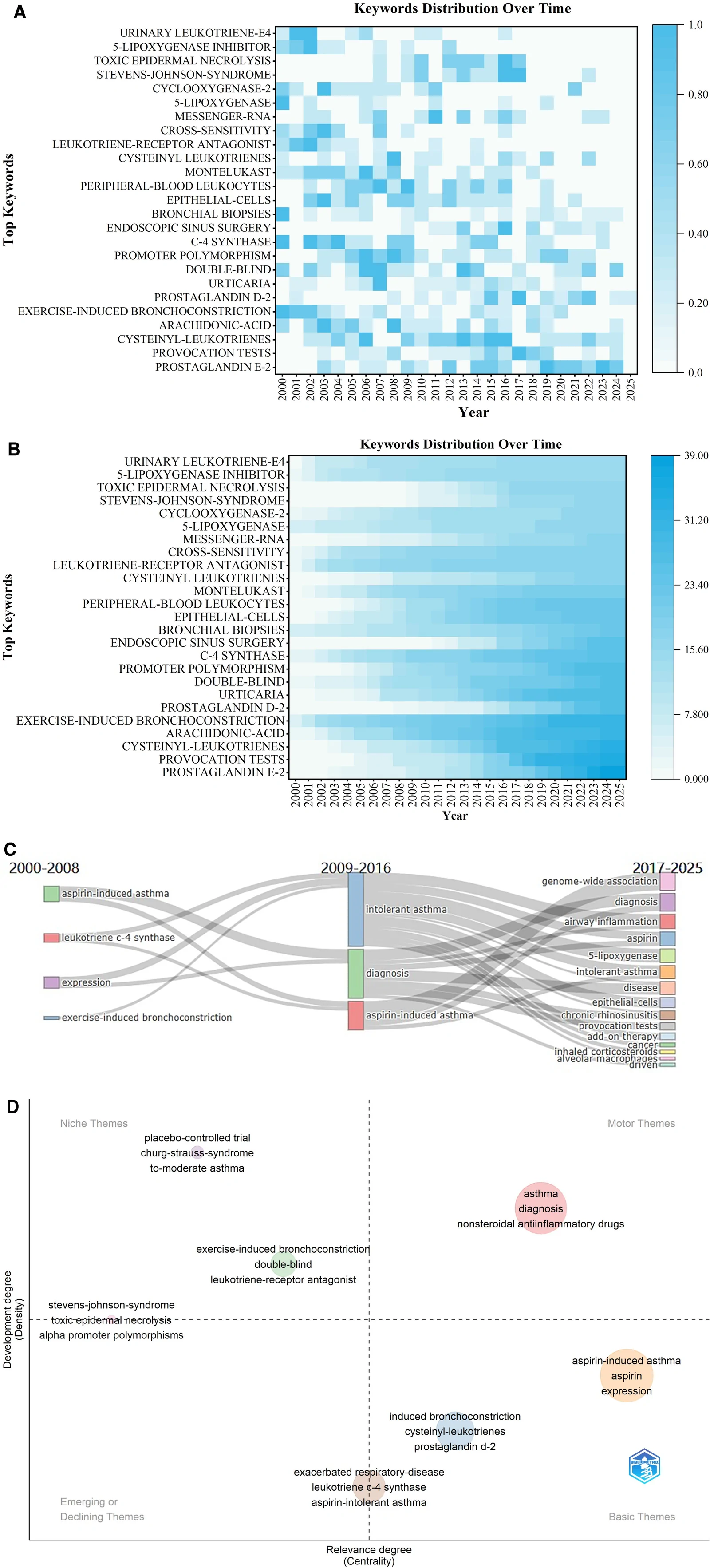
Keyword analysis of NERD. (A) Annual occurrence of keywords; (B) cumulative occurrence of keywords over time; (C) thematic evolution map of keywords; and (D) keyword cluster analysis. NERD = nonsteroidal anti-inflammatory drug (NSAID)-exacerbated respiratory disease.

### 3.7. Journal dual-map overlay analysis

The journal dual-map overlay depicts the disciplinary distribution of scholarly journals. The overlay and its visualization (Fig. 5) reveal 3 principal citation trajectories. Citing journals are predominantly located in the “medicine, medical, and clinical” and “molecular, biology, and immunology” domains, whereas cited journals are mainly concentrated in “molecular, biology, and genetics” and “health, nursing, and medicine.” Taken together, the 3 dominant citation pathways indicate that research on NERD follows a multidisciplinary, integrative development pattern.

**Figure 5.**
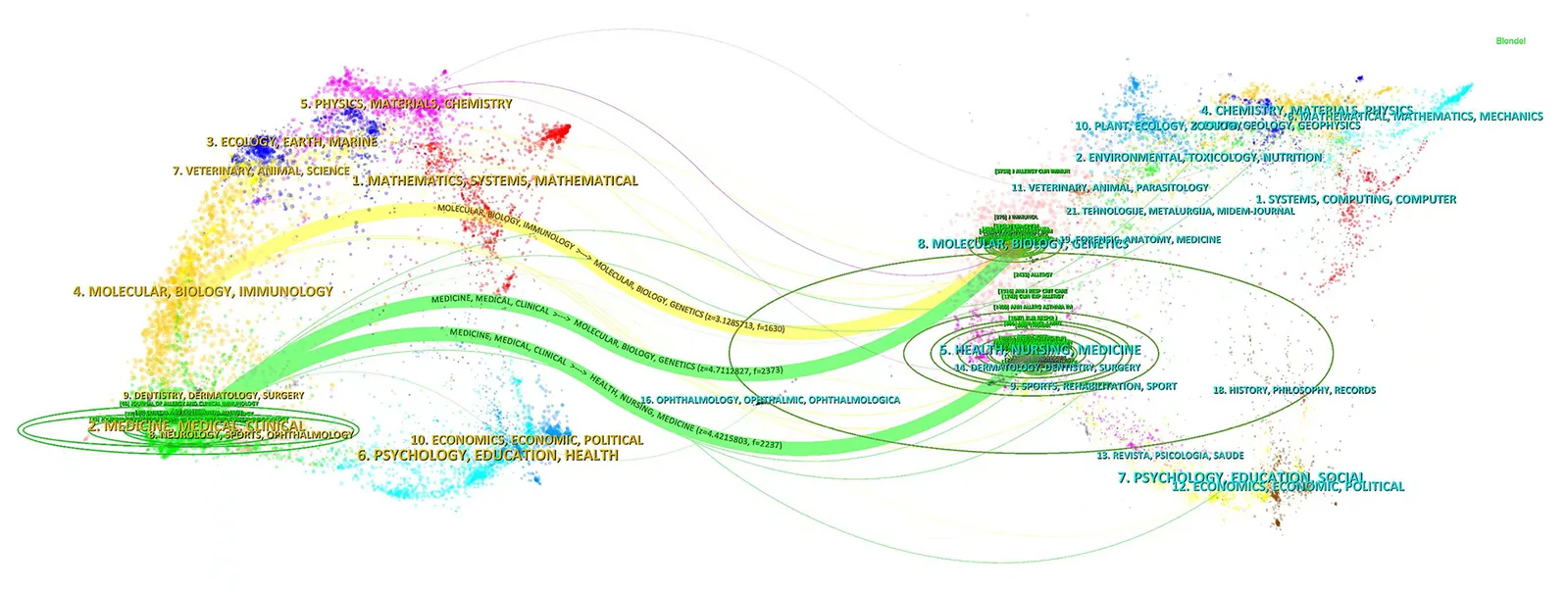
Journal dual-map overlay. Note: The figure presents a complex knowledge network. The left side represents citing journals in the field, and the right side represents clusters of cited journals. In each elliptical node, the horizontal axis length reflects the number of authors, while the vertical axis length reflects the number of published articles. The citation links in the middle connect citing and cited journals; line thickness corresponds to the frequency and strength of knowledge flow between journals.

## 4. Discussion

To our knowledge, this is the 1st bibliometric analysis of publications on NERD spanning 2000 to 2024. The items included meet the minimum requirements outlined in the preliminary guidelines for bibliometric studies. Using the WoSCC, we identified 1018 publications from January 1, 2000 to January 1, 2025, encompassing research from 195 countries, 2381 institutions, 255 journals, and 3385 authors. The analyses show a steady and sustained increase in publication volume over the past 24 years, and further growth in this field can be anticipated.

Among the top 10 countries by total output, the United States and Poland contributed the greatest number of publications; the U.S. ranked 1st, with approximately twice Poland’s output, reflecting advanced scientific capacity and substantial investment in healthcare resources. Notably, despite the high national output, only 4 U.S. institutions and 2 Polish institutions appear among the world’s top 10 most productive organizations, suggesting that NERD research in these countries is broad and diffuse across many centers, a diversified landscape that facilitates the exploration of different research directions. Notably, Jagiellonian University has shown consistently robust growth and is currently the single most productive institution in this field. In comparison, China’s average citation rate is relatively low, and no Chinese institutions appear in the high-output group, indicating that the influence and depth of related research require strengthening.

The most prolific author in the field was Marek Sanak of Jagiellonian University, whose work primarily focuses on the basic scientific aspects of NERD. His most highly cited paper is “Leukotriene C4 synthase promoter polymorphism and risk of aspirin-induced asthma”^[16]^ published in The Lancet in 1997. The top 10 journals by publication count together accounted for 31.43% of all papers in the field, underscoring their central academic role. Among the co-cited journals, the Journal of Allergy and Clinical Immunology does not have the highest impact factor but is cited far more frequently than its peers. The Lancet has the highest impact factor and is renowned for publishing high-quality clinical research. Importantly, most of the top 10 co-cited journals fall under “allergy” and “immunology,” aligning closely with the pathophysiological underpinnings of NERD and the interests of researchers in allergy and autoimmunity.

“Citation bursts” refer to keywords whose usage frequency rose sharply over a short period (Fig. S4C, Supplemental Digital Content 12). Early research emphasized basic mechanisms and diagnostic methods, such as “aspirin-induced asthma,” “challenge,” and “leukotriene receptor antagonist.” As the field progressed, more studies probed foundational layers such as “pathogenesis,” “diagnosis,” “cells,” and “expression.” In recent years, hotspots have shifted toward more specific clinical syndromes and comorbidities – “intolerant asthma,” “aspirin-exacerbated respiratory disease,” “chronic rhinosinusitis,” and “nasal polyps.” “Chronic rhinosinusitis” showed the strongest burst (29.57), with a burst period from 2017 to the present, marking it as the current focal topic. “Nasal polyps” also exhibited a strong increase (17.27) from 2019 to the present, closely paralleling the surge in interest in chronic rhinosinusitis. The related concepts “intolerant asthma” and “aspirin-exacerbated respiratory disease” demonstrated long burst periods (notably 2014–2022 for the former) and high intensities (20.22 and 11.36, respectively), indicating sustained prominence over the last decade and highlighting the topic’s ongoing value and complexity. Some keywords showed high-intensity but short-duration bursts (e.g., “pathogenesis,” 2006–2007, intensity 9.12), often signaling breakthroughs or controversies that attract wide attention. Terminology has also evolved: earlier literature frequently used “aspirin-induced asthma,” whereas later studies increasingly adopted “aspirin-exacerbated respiratory disease,” reflecting deeper understanding and greater terminological standardization. “Aspirin-induced asthma” appeared 161 times and had the highest betweenness centrality (0.75), identifying it as a core hub that spanned the research network (Table 6). The prominence of “diagnosis” and “provocation tests” underscores the persistent emphasis on diagnostic methodologies. Repeated appearances of “nonsteroidal anti-inflammatory drugs” and leukotriene-pathway terms (e.g., “cysteinyl leukotrienes” and “leukotriene receptor antagonist”) point to drug-induced mechanisms and inflammatory pathways as recurrent themes. The high and recent frequency of “chronic rhinosinusitis” (since 2013) suggests growing attention to this comorbidity in recent years.

In early work, investigations in this field were primarily centered on molecular mechanisms. With the advent of monoclonal antibody technology, immunotherapy has progressively entered clinical trials. The dual-map overlay (Fig. 5) clarifies that the research frontiers and focal points in NERD are shifting toward medicine, immunology, and clinical science. Particularly, the overlay indicated a trend from molecular biology and genetics to immunology and clinically oriented research. The visualization is based on literature published between 2000 and 2024: journals on the left represent the citing (application-oriented) domains, journals on the right represent the cited (foundational) domains, and curved bands trace citation paths. The 3 principal paths are shown in Figure 5. The yellow trajectory shows that articles originating from the molecular/biology/genetics domain mainly cited works in molecular/biology/immunology. The remaining 2 green trajectories indicate that the citing journals are concentrated in medicine/pharmacology/clinical outlets, whereas the cited journals are clustered in molecular/biology/genetics and health/nursing/medicine. Regimens that pair immunosuppressants with other agents have emerged as a particularly promising research direction in the NERD landscape. In this study, we used reference-timeline views, keyword co-occurrence and clustering, and temporal keyword distributions to delineate the evolution and hotspots of NERD research.

The heat maps in Figure 4A and B display the annual keyword counts and their cumulative frequencies over time. Mechanism-related terms such as “arachidonic acid,” “prostaglandin E2,” “prostaglandin D2,” “cysteinyl leukotrienes,” and “C4 synthase” remain blue across multiple years, indicating persistent, long-term attention; these also appear within the “Basic Themes” quadrant in Figure 4D. Taken together, these keywords map both the foundational bedrock and future trajectory of the field. Their high centrality marks them as concepts that research across NERD continually engages with, whereas their low density suggests that substantial unknowns remain, highlighting fertile grounds for deeper mechanistic inquiry and drug discovery. As the understanding of NERD pathobiology advanced, studies increasingly investigated inflammatory mediators, immune cell populations, and genetic determinants. A substantial body of work linked NERD onset to dysregulation of the arachidonic acid metabolic network. Arachidonic acid, a polyunsaturated fatty acid, is principally processed through the cyclooxygenase (COX) and lipoxygenase pathways. Prostaglandin E2 (PGE2) and the cysteinyl leukotrienes (CysLTs) are pivotal inflammatory mediators within these routes^[17]^; PGE2 exerts anti-inflammatory effects by inhibiting the activation of mast cells and eosinophils, whereas CysLTs promote inflammation and airway hyperreactivity.^[18]^ In patients with NERD, PGE2 production is reduced, whereas CysLT generation is increased. Notably, the inhibition of COX-1, an enzyme central to the COX pathway, further diminishes PGE2 synthesis in NERD,^[19]^ and CysLT levels are significantly elevated in the nasal and bronchial secretions of these patients.^[20]^ The combined effect of these mediators leads to the dysregulated activation of mast cells and eosinophils, precipitating severe respiratory symptoms.

We observed that “provocation tests” and “challenge” appear as long-standing high-frequency keywords in both the temporal distribution and clustering maps, consistent with the reference-timeline view. This pattern likely reflects clinicians’ deepening understanding of NERD and the continuous refinement of corresponding diagnostic and therapeutic strategies. In the thematic map (Fig. 4D), these terms fell within the motor theme quadrant, indicating that the diagnosis of NERD constitutes the core of current research activity. The diagnosis of NERD is based on the patient’s medical history and confirmed by an aspirin provocation test before initiating treatment.^[21]^ Such challenges enabled the effective clinical identification of NERD, particularly in patients with atypical symptoms or unclear histories. Principal modalities include the oral aspirin challenge (OAC), intranasal aspirin challenge (IAC), and bronchial aspirin challenge.^[22,23]^ OAC was the most commonly used and involved stepwise dose escalation of aspirin while monitoring respiratory symptoms (e.g., nasal obstruction and bronchospasm). IAC involves the local application of aspirin or NSAIDs to assess nasal mucosal reactivity and offers high specificity and sensitivity. The bronchial aspirin challenge evaluates airway hyperresponsiveness via inhaled aspirin and is useful when NERD is suspected; however, the results of OAC are inconclusive. Earlier challenge protocols carried substantial risks, with the potential for severe respiratory reactions and life-threatening events. As research has advanced and clinical guidelines have matured, protocols have become more standardized and safe; under rigorous monitoring, the incidence of serious adverse events during OAC can be markedly reduced. Owing to lower doses and purely local administration, IAC confers a more favorable safety profile and is suitable for high-risk patients.^[24]^ Low-dose aspirin challenges are increasingly adopted; most patients manifested positive responses at doses ≤162 mg, thereby achieving diagnostic yield while limiting adverse effects.^[25,26]^

In recent years, aspirin therapy after desensitization (ATAD) has become the 1st-line therapy for NERD, characterized by its increasing clinical adoption and broadly validated efficacy. ATAD induces tolerance to aspirin by gradually escalating the dose, thereby restoring PGE_2_ production and suppressing excessive generation of CysLTs, which in turn mitigates aspirin-triggered inflammation. ATAD comprises 2 sequential phases: an initial desensitization phase, typically completed within 2 to 3 days, followed by a long-term maintenance phase lasting at least 6 months; some patients require lifelong therapy to achieve adequate symptom control. ATAD improves symptoms, reduces the frequency of nasal polyp recurrence and asthma exacerbations, and, after sustained treatment, enhances olfactory function and overall quality of life in patients with NERD.^[27]^

The timeline visualization of references showed that research on immunotherapy became a prominent topic in recent years. A dual-map overlay of journals, keyword co-occurrence, and clustering analysis has identified this field as a hotspot. Immunotherapeutic agents can significantly improve nasal and bronchial symptoms in patients with NERD, reduce nasal polyp recurrence and the frequency of acute asthma exacerbations, and improve olfactory function and quality of life, particularly with long-term treatment. These studies provide important clinical evidence for the use of immunotherapeutic agents in NERD, indicating that such treatments not only alleviate asthma symptoms but also reduce nasal polyps and chronic rhinosinusitis. Future research should focus on the combined use of immunotherapeutic agents, as combination therapy can target different immune pathways to provide a more comprehensive immunomodulatory effect, thereby enhancing the efficacy of NERD treatment. The combined use of these agents may yield synergistic effects through different mechanisms, offering stronger immunomodulation and potentially benefiting patients who do not respond well to a single immunotherapeutic agent. Thymic stromal lymphopoietin (TSLP) is a cytokine secreted by various epithelial cells (especially airway epithelial cells) under chronic inflammatory or injury stimuli; it plays a key role in allergic immune responses.^[28]^ In patients with NERD, TSLP binds to its TSLP receptor and activates type 2 innate lymphoid cells (ILC2s) and mast cells, promoting a type 2 inflammatory response that exacerbates NERD symptoms. Thymic stromal lymphopoietin can also enhance interleukin-4 receptor alpha signaling, thereby further aggravating airway inflammation.^[29,30]^ In recent years, monoclonal antibodies targeting TSLP (such as tezepelumab) have shown potential clinical value; by binding to TSLP and blocking its interaction with the receptor, they inhibit downstream inflammatory responses.^[31–33]^ Thus, anti-TSLP monoclonal antibodies represent an emerging trend in immunotherapeutic research. However, current research is limited, and more studies are needed to support their clinical efficacy. IL-33 is an immunostimulatory cytokine that acts in concert with other cytokines (like TSLP) to activate ILC2s and mast cells, driving type 2 inflammation and worsening airway inflammation.^[34,35]^ Blocking the IL-33 signaling pathway may reduce type 2 inflammation by inhibiting the activation of mast cells and ILC2s. Therapeutic strategies targeting IL-33 may provide new treatment directions for NERD; however, additional clinical studies are required to verify their safety and efficacy. These new targeted therapeutic approaches currently show considerable promise for NERD. Future clinical studies will help confirm their efficacy and explore how to integrate them with other treatments to further improve patient outcomes.

Pharmacotherapy remains the mainstay in the management of NERD; however, surgical intervention is an important option for patients who fail to respond adequately to pharmacotherapy or experience recurrent nasal polyps. Figure 4A shows that endoscopic sinus surgery has become an area of increasing research interest in recent years. The primary objectives of surgery are to improve nasal airflow, reduce inflammatory burden, and optimize conditions for pharmacological treatment; however, the high rate of revision surgery due to polyp recurrence poses a major challenge in the long-term management of NERD. As the pathophysiology of NERD is further elucidated, surgical treatment strategies continue to evolve. Surgery not only removes nasal polyps and reduces inflammatory cell infiltration, thereby lowering inflammatory mediator levels, but also improves nasal mucosal epithelial barrier function, limiting the release of these mediators and further alleviating symptoms.^[36,37]^

Alcohol, a common dietary component, exerts immunomodulatory effects through its metabolites (such as acetaldehyde), which can influence airway inflammation via multiple pathways. It is recognized as a frequent non-pharmacological trigger in patients with NERD and can induce or exacerbate symptoms, likely through mechanisms involving mast cell activation and leukotriene release. Approximately 77% of patients with NERD experience nasal congestion and worsening of asthma symptoms after alcohol consumption, and most patients identify alcohol as a potential trigger for their disease.^[38]^ In contrast, only 3.4% to 7.4% of the general population reports alcohol-induced nasal symptoms.^[39]^ A double-blind, placebo-controlled crossover trial demonstrated that pretreatment with montelukast and cetirizine significantly attenuated alcohol-induced respiratory symptoms in NERD, suggesting a pivotal role for leukotrienes and histamine in mediating these responses.^[40]^ Reducing alcohol intake can substantially decrease symptom exacerbation and disease progression in NERD, and patients undergoing aspirin desensitization therapy are advised to limit alcohol consumption to minimize the risk of disease deterioration. Therefore, alcohol contributes to enhanced airway inflammation and symptom burden in NERD through multiple mechanisms closely related to mast cell activation and release of inflammatory mediators, although the precise pathways remain to be elucidated and warrant further investigation.

Owing to the insidious and complex nature of NERD symptoms and their frequent overlap with other respiratory diseases, early diagnosis and intervention remain both a clinical priority and a persistent challenge. Early screening and intervention not only help delay disease progression but also markedly improve patients’ quality of life. Initial screening for NERD largely relies on clinical symptoms and patient history; however, in the early stages, these manifestations are often atypical and can easily be misdiagnosed as common asthma or chronic rhinosinusitis. Therefore, clinicians typically employ diagnostic tools, such as aspirin challenge tests, in combination with clinical assessments to identify individuals at high risk for NERD.

Given the diversity of pathophysiological mechanisms and the heterogeneity of clinical manifestations of NERD, its treatment remains a significant clinical challenge, and individualized therapeutic strategies have gradually emerged as a research focus. Personalized treatment aims to tailor therapeutic regimens according to patients’ inflammatory phenotypes, genetic characteristics, and clinical responses, thereby enhancing efficacy while minimizing adverse effects. Biomarkers such as urinary leukotriene E4 level,^[41]^ peripheral blood eosinophil counts, and leukotriene levels are associated with disease severity and treatment responsiveness in patients with NERD. Monitoring the changes in these biomarkers may assist clinicians in evaluating therapeutic efficacy and adjusting treatment strategies in a timely manner. Moreover, advances in genomics have opened new possibilities for individualized management by enabling genetic profiling. For example, specific genetic variants may influence aspirin tolerance or responsiveness to immunotherapeutic agents, suggesting that genetic testing could help in designing more personalized therapeutic approaches in the future.

Future research is likely to focus on integrating precision medicine with individualized treatments to achieve personalized NERD management through more accurate immune monitoring, genetic analysis, and biomarker profiling. Notably, “add-on therapy” has emerged as a frequently cited keyword in recent years, which reflects the evolution of therapeutic strategies. Rather than relying solely on conventional approaches, investigators are increasingly exploring combination regimens that integrate immunotherapeutics, small-molecule agents, and traditional treatments, which are expected to become a mainstream trend in NERD management. Through multidisciplinary collaboration and the integration of insights from immunology, genomics, and pharmacology, future individualized treatment strategies are anticipated to become more precise, ultimately providing patients with NERD with more effective therapeutic options.

The bibliometric trends identified in this study also reflect important changes in the clinical management of NERD. The increasing prominence of aspirin desensitization, biologic therapy, and endoscopic sinus surgery (ESS) indicates a transition from predominantly mechanism-oriented research toward multidisciplinary and phenotype-directed clinical management. Current guidelines and expert recommendations support ATAD as a therapeutic option for appropriately selected patients with NERD. TSLP represents an upstream epithelial target of particular interest. Recent clinical evidence has demonstrated the efficacy of the anti-TSLP monoclonal antibody tezepelumab in severe CRSwNP, supporting the translational importance of TSLP-related research. However, evidence specifically derived from NERD populations remains comparatively limited. IL-33 is also biologically relevant to mast-cell and ILC2 activation in NERD, but IL-33 targeted therapy remains an emerging strategy and requires further clinical validation before it can be considered an established component of NERD management. ESS remains an important component of treatment for patients with persistent or recurrent disease despite appropriate medical therapy. Surgery can reduce the inflammatory burden, restore sinus ventilation, and improve access for topical postoperative therapies. Current clinical management increasingly emphasizes individualized and multimodal treatment in which surgery, ATAD, biologic therapy, and conventional pharmacotherapy may be used sequentially or in combination according to disease severity, asthma control, previous treatment response, safety considerations, and patient preferences.

Several limitations of this study should be acknowledged. First, although the WoSCC provides standardized citation data and is widely used in bibliometric analyses, reliance on a single database may have introduced database-related selection bias. Relevant publications indexed exclusively in other databases, such as Scopus or PubMed, may therefore have been omitted. Differences in database coverage, indexing policies, and citation records could potentially influence the identified distributions of countries, institutions, authors, journals, keywords, and citation networks. Second, only English-language publications were included in this analysis. This restriction may have resulted in the underrepresentation of relevant studies published in other languages, particularly those originating from European and Asian countries. Therefore, the findings of the present study should be interpreted as primarily reflecting the structure and research trends of the English-language literature indexed in WoSCC, rather than the entirety of global research in this field.

In conclusion, by employing bibliometric visualization tools, such as CiteSpace and VOSviewer, this study systematically analyzed and visualized research on NERD over the past 24 years. It comprehensively summarizes the hotspots, developmental trajectories, and emerging trends in this field. These findings indicate that the United States has made substantial contributions, whereas Asian countries need to strengthen their production of high-quality research. Moreover, the results highlight the necessity of fostering multilevel collaboration across countries, regions, institutions, and researchers. The observed trends suggest that future research should gradually shift focus from molecular biology and genetics to immunology and clinical studies, with individualized therapy ultimately emerging as a key direction for the management of NERD. Clinically, the increasing prominence of ATAD, biologic therapy, and ESS reflects a transition toward multimodal and individualized management of NERD. Rather than representing mutually exclusive approaches, these treatments should be selected or combined according to the severity of upper- and lower-airway disease, previous treatment response, comorbidities, safety considerations, and patient preferences. Future research should further define biomarkers and clinical criteria that can guide treatment selection and optimize outcomes in patients with NERD.

## Author contributions

**Data curation:** Dayu Guan, Yuan Chen, Yalan Zhang, Ji Wang, Yue Gu, Bowen Zheng, Yuchuan Liu, Xiaoling Zhang.

**Funding acquisition:** Chenxi Li, Yucheng Yang.

**Software:** Dayu Guan.

**Supervision:** Tao Lu, Yang Shen, Yucheng Yang.

**Visualization:** Dayu Guan.

**Writing – original draft:** Chenxi Li.

**Writing – review & editing:** Chenxi Li.

























## References

[1] Andersén H Ilmarinen P Honkamäki J. NSAID-exacerbated respiratory disease: a population study. ERJ Open Res. 2022;8:00462–2021. 10.1183/23120541.00462-2021 PMC8784895 35083326

[2] Leroyer C Eraso I Sangiovanni S Morales EI Fernández-Trujillo L. Aspirin desensitization in NSAID-exacerbated respiratory disease and its outcomes in the clinical course of asthma: a systematic review of the literature and meta-analysis. PLoS One. 2021;16:e0247871. 10.1371/journal.pone.0247871 PMC7996991 33770090

[3] Cockcroft DW. NSAID-exacerbated respiratory disease, dupilumab and aspirin tolerance. Eur Respir J. 2023;61:2202467. 10.1183/13993003.02467-2022 36927861

[4] Ninkov A Frank JR Maggio LA. Bibliometrics: methods for studying academic publishing. Perspect Med Educ. 2022;11:173–6. 10.1007/s40037-021-00695-4 PMC9240160 34914027

[5] Agarwal A Durairajanayagam D Tatagari S. Bibliometrics: tracking research impact by selecting the appropriate metrics. Asian J Androl. 2016;18:296–309. 10.4103/1008-682X.171582 PMC4770502 26806079

[6] Szczeklik A Nizankowska E Duplaga M. Natural history of aspirin-induced asthma. AIANE Investigators. European Network on Aspirin-Induced Asthma. Eur Respir J. 2000;16:432–6. 10.1034/j.1399-3003.2000.016003432.x 11028656

[7] Cowburn AS Sladek K Soja J. Overexpression of leukotriene C4 synthase in bronchial biopsies from patients with aspirin-intolerant asthma. J Clin Invest. 1998;101:834–46. 10.1172/JCI620 PMC508632 9466979

[8] Szczeklik A Stevenson DD. Aspirin-induced asthma: advances in pathogenesis, diagnosis, and management. J Allergy Clin Immunol. 2003;111:913–21; quiz 922. 10.1067/mai.2003.1487 12743549

[9] Samter M Beers RF. Intolerance to aspirin. Clinical studies and consideration of its pathogenesis. Ann Intern Med. 1968;68:975–83. 10.7326/0003-4819-68-5-975 5646829

[10] Christie PE Tagari P Ford-Hutchinson AW. Urinary leukotriene E4 concentrations increase after aspirin challenge in aspirin-sensitive asthmatic subjects. Am Rev Respir Dis. 1991;143(5 Pt 1):1025–9. 10.1164/ajrccm/143.5_Pt_1.1025 1850964

[11] Berges-Gimeno MP Simon RA Stevenson DD. The natural history and clinical characteristics of aspirin-exacerbated respiratory disease. Ann Allergy Asthma Immunol. 2002;89:474–8. 10.1016/S1081-1206(10)62084-4 12452205

[12] Nizankowska-Mogilnicka E Bochenek G Mastalerz L. EAACI/GA2LEN guideline: aspirin provocation tests for diagnosis of aspirin hypersensitivity. Allergy. 2007;62:1111–8. 10.1111/j.1398-9995.2007.01409.x 17521312

[13] Szczeklik A Stevenson DD. Aspirin-induced asthma: advances in pathogenesis and management. J Allergy Clin Immunol. 1999;104:5–13. 10.1016/s0091-6749(99)70106-5 10400832

[14] Jenkins C Costello J Hodge L. Systematic review of prevalence of aspirin induced asthma and its implications for clinical practice. BMJ. 2004;328:434. 10.1136/bmj.328.7437.434 PMC344260 14976098

[15] Sousa AR Parikh A Scadding G Corrigan CJ Lee TH. Leukotriene-receptor expression on nasal mucosal inflammatory cells in aspirin-sensitive rhinosinusitis. N Engl J Med. 2002;347:1493–9. 10.1056/NEJMoa013508 12421891

[16] Sanak M Simon HU Szczeklik A. Leukotriene C4 synthase promoter polymorphism and risk of aspirin-induced asthma. Lancet. 1997;350:1599–600. 10.1016/s0140-6736(05)64015-9 9393345

[17] Laidlaw TM Boyce JA. Updates on immune mechanisms in aspirin-exacerbated respiratory disease. J Allergy Clin Immunol. 2023;151:301–9. 10.1016/j.jaci.2022.08.021 PMC9905222 36184313

[18] Mastalerz L Tyrak KE Ignacak M. Prostaglandin E2 decrease in induced sputum of hypersensitive asthmatics during oral challenge with aspirin. Allergy. 2019;74:922–32. 10.1111/all.13671 30446997

[19] Laidlaw TM Boyce JA. Platelets in patients with aspirin-exacerbated respiratory disease. J Allergy Clin Immunol. 2015;135:1407–14; quiz 1415. 10.1016/j.jaci.2015.02.005 PMC4559084 26051947

[20] Taniguchi M Mitsui C Hayashi H. Aspirin-exacerbated respiratory disease (AERD): current understanding of AERD. Allergol Int. 2019;68:289–95. 10.1016/j.alit.2019.05.001 31235242

[21] Kowalski ML Agache I Bavbek S. Diagnosis and management of NSAID-exacerbated respiratory disease (NERD) – a EAACI position paper. Allergy. 2019;74:28–39. 10.1111/all.13599 30216468

[22] Lourijsen E Avdeeva K Gan KL Fokkens W. Oral and intranasal aspirin desensitisation for non-steroidal anti-inflammatory drug (NSAID)-exacerbated respiratory disease. Cochrane Database Syst Rev. 2025;1:CD013476. 10.1002/14651858.CD013476.pub2 PMC12043203 39775459

[23] Sánchez-Jareño M Barranco P Padial Vilchez MA. Changes in fractional exhaled nitric oxide levels after bronchial challenge with aspirin in patients with aspirin-induced asthma. J Investig Allergol Clin Immunol. 2019;29:137–9. 10.18176/jiaci.0344 31017112

[24] Nguyen A Zuraw BL Wu C Kim A Christiansen SC. Intranasal ketorolac, diagnosis, and desensitization for aspirin-exacerbated respiratory disease. Ann Allergy Asthma Immunol. 2021;126:674–80. 10.1016/j.anai.2021.01.011 33476718

[25] DeGregorio GA Singer J Cahill KN Laidlaw T. A 1-Day, 90-minute aspirin challenge and desensitization protocol in aspirin-exacerbated respiratory disease. J Allergy Clin Immunol Pract. 2019;7:1174–80. 10.1016/j.jaip.2018.10.032 PMC6456426 30391549

[26] Pelletier T Roizen G Ren Z Hudes G Rosenstreich D Jerschow E. Comparable safety of 2 aspirin desensitization protocols for aspirin exacerbated respiratory disease. J Allergy Clin Immunol Pract. 2019;7:1319–21. 10.1016/j.jaip.2018.08.004 30144584

[27] Helevä A Lyly A Salmi V. Efficacy of aspirin therapy after desensitization on chronic rhinosinusitis with nasal polyps patients with asthma and non-steroidal anti-inflammatory drug exacerbated respiratory disease – a randomized clinical trial. Allergy. 2025;80:3151–64. 10.1111/all.16679 PMC12590337 40726272

[28] Klimek L Hagemann J Welkoborsky H-J. Epithelial immune regulation of inflammatory airway diseases: chronic rhinosinusitis with nasal polyps (CRSwNP). Allergol Select. 2022;6:148–66. 10.5414/ALX02296E PMC9097524 35572064

[29] Boita M Garzaro M Raimondo L. The expression of TSLP receptor in chronic rhinosinusitis with and without nasal polyps. Int J Immunopathol Pharmacol. 2011;24:761–8. 10.1177/039463201102400322 21978707

[30] Nagarkar DR Poposki JA Tan BK. Thymic stromal lymphopoietin activity is increased in nasal polyps of patients with chronic rhinosinusitis. J Allergy Clin Immunol. 2013;132:593–600.e12. 10.1016/j.jaci.2013.04.005 PMC3759596 23688414

[31] Emson C Corren J Sałapa K Hellqvist A Parnes JR Colice G. Efficacy of tezepelumab in patients with severe, uncontrolled asthma with and without nasal polyposis: a post hoc analysis of the phase 2b PATHWAY study. J Asthma Allergy. 2021;14:91–9. 10.2147/JAA.S288260 PMC7868291 33568920

[32] Nolasco S Pelaia C Scioscia G Campisi R Crimi C. Tezepelumab for asthma. Drugs Today (Barc). 2022;58:591–603. 10.1358/dot.2022.58.12.3449205 36651067

[33] Pelaia C Pelaia G Crimi C. Tezepelumab: a potential new biological therapy for severe refractory asthma. Int J Mol Sci. 2021;22:4369. 10.3390/ijms22094369 PMC8122263 33922072

[34] Allakhverdi Z Smith DE Comeau MR Delespesse G. Cutting edge: the ST2 ligand IL-33 potently activates and drives maturation of human mast cells. J Immunol. 2007;179:2051–4. 10.4049/jimmunol.179.4.2051 17675461

[35] Pan D Buchheit KM Samuchiwal SK. COX-1 mediates IL-33-induced extracellular signal-regulated kinase activation in mast cells: implications for aspirin sensitivity. J Allergy Clin Immunol. 2019;143:1047–57.e8. 10.1016/j.jaci.2018.06.033 PMC6330164 30017554

[36] Loftus CA Soler ZM Koochakzadeh S. Revision surgery rates in chronic rhinosinusitis with nasal polyps: meta-analysis of risk factors. Int Forum Allergy Rhinol. 2020;10:199–207. 10.1002/alr.22487 31752051

[37] Adelman J McLean C Shaigany K Krouse JH. The role of surgery in management of samter’s triad: a systematic review. Otolaryngol Head Neck Surg. 2016;155:220–37. 10.1177/0194599816640723 27071444

[38] Ta V White AA. Survey-defined patient experiences with aspirin-exacerbated respiratory disease. J Allergy Clin Immunol Pract. 2015;3:711–8. 10.1016/j.jaip.2015.03.001 25858054

[39] De Schryver E Derycke L Campo P. Alcohol hyper-responsiveness in chronic rhinosinusitis with nasal polyps. Clin Exp Allergy. 2017;47:245–53. 10.1111/cea.12836 27770460

[40] Ramos CL Thakrar H Walters K Waldram J White AA. Characterization of alcohol-induced respiratory symptoms in aspirin-exacerbated respiratory disease. Ann Allergy Asthma Immunol. 2023;131:382–4. 10.1016/j.anai.2023.06.002 PMC10528382 37302527

[41] Szatkowski P Stępień A Ćmiel A. Metabolomic endophenotypes based on oxo-eicosatetraenoic acids in patients with aspirin-exacerbated respiratory disease. J Allergy Clin Immunol. 2025;156:616–26. 10.1016/j.jaci.2025.04.036 40449668

